# Optimized microRNA purification from TRIzol-treated plasma

**DOI:** 10.1186/s12864-015-1299-5

**Published:** 2015-02-18

**Authors:** Janice Duy, Jeffrey W Koehler, Anna N Honko, Timothy D Minogue

**Affiliations:** Diagnostic Systems Division, U.S. Army Medical Research Institute of Infectious Diseases, 1425 Porter St., Fort Detrick, Frederick, MD 21701 USA; Integrated Research Facility, National Institute of Allergy and Infectious Diseases, National Institutes of Health, 8200 Research Plaza, Fort Detrick, Frederick, MD 21701 USA; Virology Division, U.S. Army Medical Institute of Infectious Diseases, 1425 Porter St., Fort Detrick, Frederick, MD 21701 USA

**Keywords:** microRNA, TRIzol, Ebola virus, RNA extraction, Plasma, Biomarker, RT-PCR, Nonhuman primate

## Abstract

**Background:**

MicroRNAs (miRNAs) represent new and potentially informative diagnostic targets for disease detection and prognosis. However, little work exists documenting the effect of TRIzol, a common viral inactivation and nucleic acid extraction reagent, on miRNA purification. Here, we developed an optimized protocol for miRNA extraction from plasma samples by evaluating five different RNA extraction kits, TRIzol phase separation, purification additives, and initial plasma sample volume. This method was then used for downstream profiling of plasma miRNAs found in archived samples from one nonhuman primate (NHP) experimentally challenged with Ebola virus by the aerosol route.

**Results:**

Comparison of real-time RT-PCR results for spiked-in and endogenous miRNA sequences determined extraction efficiencies from five different RNA purification kits. These experiments showed that 50 μL plasma processed using the QIAGEN miRNeasy Mini Kit with 5 μg of glycogen as a co-precipitant yielded the highest recovery of endogenous miRNAs. Using this optimized protocol, miRNAs from archived plasma samples of one rhesus macaque challenged with aerosolized Ebola virus was profiled using a targeted real-time PCR array. A total of 519 of the 752 unique miRNAs assayed were present in the plasma samples at day 0 and day 7 (time of death) post-exposure. Statistical analyses revealed 25 sequences significantly up- or down-regulated between day 0 and day 7 post infection, validating the utility of the extraction method for plasma miRNA profiling.

**Conclusions:**

This study contributes to the knowledgebase of circulating miRNA extraction methods and expands on the potential applications of cell-free miRNA profiling for diagnostics and pathogenesis studies. Specifically, we optimized an extraction protocol for miRNAs from TRIzol-inactivated plasma samples that can be used for highly pathogenic viruses.

**Electronic supplementary material:**

The online version of this article (doi:10.1186/s12864-015-1299-5) contains supplementary material, which is available to authorized users.

## Background

MicroRNA (miRNA) profiling from plasma or serum is emerging as a minimally invasive source of disease-specific biomarker signatures (reviewed in [1]). Unlike other nucleic acids, circulating miRNAs are resistant to degradation [2-4] and can be differentially expressed according to disease state (reviewed in [5,6]). Since the relatively low numbers of miRNAs may simultaneously regulate disparate gene networks [7], miRNA patterns can be easier to analyze than global gene signatures. However, accurately identifying circulating miRNA disease biomarkers is highly dependent on pre-analytical variables such as sample type and collection, processing, storage, and RNA isolation method (reviewed in [6]). For instance, recent studies have demonstrated that standard (platelet-replete) plasma and hemolysis can mask true disease biomarkers through elevated blood cell-derived miRNA levels [8-11].

Characterizing host-encoded miRNAs differentially observed following exposure to highly infectious category A pathogens such as Ebola virus, Lassa fever virus, or *Bacillus anthracis* could be used to prevent disease transmission and improve patient outcomes. However, biomarker discovery from these agents is complicated by the limited number of animal studies available and by highly restricted access to these samples. In addition, these pathogens must be inactivated by radiological, thermal, or chemical means before removal from biocontainment suites [12]. Of these methods, chemical inactivation with a guanidinium-phenol-based solution such as TRIzol/TRI Reagent or equivalent is convenient for viruses as it works immediately, does not require specialized equipment, and is compatible with multiple tissue types [13]. TRIzol is also routinely used for RNA isolation from clinical samples with alcohol precipitation, but overnight incubation and extended centrifugation are recommended to enhance miRNA yield [14]. More rapid column-based purification kits evolved from this method, and historically most miRNA studies have used either the Ambion *mir*Vana or QIAGEN miRNeasy kits (reviewed in [6]). Samples can also be lysed with TRIzol and purified via spin column in a hybrid approach [3]. However, inconsistencies in miRNA yield and sample-to-sample variance, dependent on RNA extraction, have been reported [10,15,16]. Also, at low cell densities, miRNA isolation using TRIzol significantly decreased the recovery of sequences with stable secondary structure and low GC content [17]. Consequently, extraction methodologies must be optimized for each sample type and application [18].

In this study, we initially evaluated five commercially available spin column-based RNA isolation kits for purification of TRIzol-inactivated nonhuman primate (NHP) plasma samples. RNA recovery was assessed using RT-PCR assays targeting synthetic spike-in RNA sequences as well as selected endogenous miRNAs. Glycogen and linear acrylamide were investigated as nucleic acid co-precipitants since RNA yields from cell-free samples such as plasma are low [7]. Minimum plasma volumes were determined for miRNome expression profiling, and optimized extraction parameters were used in a pilot study utilizing archived plasma samples from one NHP infected with Ebola virus.

## Results

### Healthy nonhuman primate (NHP) plasma sample characterization

Previous studies have shown that blood cell contamination can artificially inflate miRNA expression levels [8-11,15,19], so plasma samples must be tested for hemolysis. For the RNA extraction protocols tested here, we used a pooled plasma sample from three healthy NHPs. Two successive centrifugation steps removed the majority of platelets in the samples; a hematology analyzer measured the platelet count of twice-centrifuged plasma as 6,930 cells/μL, and hemoglobin content at 0.05 g/L. These values indicate that the plasma sample was platelet-poor and not hemolyzed. Lack of hemolysis-dependent miRNA elevation suggested minimal blood cell contamination and negligible impact on miRNA expression changes.

### QIAGEN kits yielded best miRNA recovery

Selective elution of RNA < 200 nt (small RNA enrichment) can reduce miRNA recovery and introduce experimental bias [20], so manufacturers’ protocols were modified for optimal isolation of total RNA (Additional file 1: Supplementary methods). Since extracted RNA could not be measured spectrophotometrically or with an Agilent BioAnalyzer (data not shown), kit performance was evaluated using individual miRCURY LNA real-time PCR assays (Exiqon, Inc.). We used three Exiqon synthetic spike-in sequences, designed to mimic miRNAs with short sequence lengths and GC content ranging from 43-50% [21], for preliminary evaluation of miRNA recovery. In addition, we chose seven endogenous miRNA sequences based on previously published data shown in Table 1. Specifically, these sequences showed consistent abundance and persistence in plasma samples. Both blood cell-derived [11,22] and circulating species were selected to assess miRNA recovery.

**Table 1:** **Individual endogenous miRNAs assayed using RT-PCR**

**MicroRNA** ^**a**^	**Blood cell-derived** ^**b**^	**Cq from** **[** 8 **]** ^**c**^
hsa-miR-451a	Yes - RBC [11]	22.5
hsa-miR-16-5p	Yes - RBC [11]	23.2
hsa-miR-93-5p	No	27.2
hsa-miR-23a-3p	Yes – platelet [22]	27.9
hsa-miR-423-3p	Yes – platelet [22]	30.9
hsa-miR-103a-3p	Yes – platelet [22]	28.6
hsa-miR-205-5p	No	34

^**a**^miRBase release 21 nomenclature.

^**b**^Based on current literature.

^**c**^Cq values obtained from 200 μL plasma assayed using the Exiqon Ready-to-Use PCR Human panel I V1.M. Values given are for platelet-poor plasma samples.

Monitoring extraction of these seven endogenous and three spike-in sequences extracted from 10 separate aliquots of the pooled plasma sample showed increased RNA yields from the QIAGEN RNeasy and miRNeasy kits compared to the other three protocols tested (Table 2). On average, these kits enabled miRNA detection at 7.4, 2.8, and 2.0 cycles before the Ambion, Exiqon, and Zymo kits, respectively (linear mixed effects model, p < 0.0001). RNA extracted using the QIAGEN kits also resulted in detection of the seven miRNA sequences in 10 of 10 technical replicates. In contrast, the other kits demonstrated varying recoveries of lower-abundance miRNA species. Of note, the Ambion *mir*Vana kit resulted in UniSp5 detection in 3 of 10 technical replicates, and hsa-miR-205-5p was not detected in any plasma samples. Thus, we downselected to the QIAGEN RNeasy and miRNeasy kits for subsequent testing.

**Table 2:** **MiRNA recovery by kit, presented as average Cq ± SD (n = 10)**

**RNA Target**	**Ambion** ***miR*** **vana miRNA Isolation Kit**	**Exiqon RNA Isolation Kit -Biofluids**	**QIAGEN RNeasy Mini Kit**	**QIAGEN miRNeasy Mini Kit**	**Zymo Direct-zol RNA MiniPrep Kit**
UniSp2	28.6 ± 0.3	24.9 ± 0.4	21.8 ± 0.4	21.5 ± 0.3	23.7 ± 0.5
UniSp4	34.8 ± 0.9	31.9 ± 0.6	28.9 ± 0.8	28.7 ± 0.8	30.9 ± 0.4
UniSp5	**33.7 ± 6.0(3)**	**36.4 ± 0.4(7)**	34.7 ± 1.4	34.1 ± 2.0	**38.3 ± 3.6(8)**
hsa-miR-451a	30.6 ± 0.4	26.8 ± 0.5	23.7 ± 0.3	23.6 ± 0.4	25.2 ± 0.5
hsa-miR-16-5p	31.3 ± 0.4	27.6 ± 0.5	24.7 ± 0.4	24.8 ± 0.5	26.6 ± 0.5
hsa-miR-93-5p	34.6 ± 0.5	31.0 ± 0.5	28.4 ± 0.4	28.5 ± 0.4	30.3 ± 0.5
hsa-miR-23a-3p	35.8 ± 0.6	31.9 ± 0.4	29.6 ± 0.4	29.5 ± 0.5	31.3 ± 0.6
hsa-miR-423-3p	**40.6 ± 4.7(4)**	33.1 ± 0.8	31.1 ± 0.4	31.1 ± 0.4	33.2 ± 0.4
hsa-miR-103a-3p	**38.9 ± 1.5(8)**	33.9 ± 0.5	31.5 ± 0.3	31.5 ± 0.5	32.2 ± 1.2
hsa-miR-205-5p	**NA(0)**	**36.5 ± 0.5(9)**	35.5 ± 0.6	35.6 ± 0.6	38.1 ± 1.9

Numbers in parentheses represent numbers of samples where miRNA was recovered, if less than 10 of 10 technical replicates. These entries are in bold for convenience.

### Phase separation decreases sample-to-sample variance

**Figure 1:**
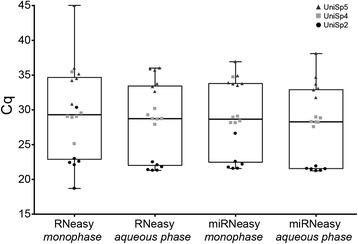
**Effect of phase separation on miRNA recovery using QIAGEN spin column-based purification kits.** The recovery of three different amounts of spike-in sequences (UniSp2, circles; UniSp4, squares; UniSp5, triangles) was compared by real-time RT-PCR for unspun TRIzol-plasma lysate (monophase) and TRIzol-plasma aqueous phase as kit inputs. Boxplots represent aggregated Cq values from the three spike-ins, with whiskers at the minimum and maximum values, and the line drawn at the median. Individual datapoints are included to show spread (n = 6 for each sequence; total n = 18). While the difference in yields was not statistically significant, using the aqueous phase showed a trend of decreased sample-to-sample variance.

Separation of the aqueous phase after TRIzol lysis may lead to reduction in miRNA yields due to extra sample manipulation and possible sample loss. Since the Zymo Direct-zol kit does not require phase separation prior to column purification, we investigated non-centrifuged (monophase) plasma-TRIzol lysate as input to the spin columns. A comparison of the recovery of the spike-in sequences UniSp2, UniSp4, and UniSp5 showed no statistically significant differences in the means obtained for the two QIAGEN kits (Figure 1). However, UniSp5 was not detected in 1 of 6 monophase samples obtained with the RNeasy kit. Also, we observed a trend of lower measurement variability with extractions using the aqueous phase (Figure 1, individual data points), so subsequent tests used this as input to the two QIAGEN kits.

### Addition of glycogen as co-precipitant improves miRNA recovery

**Figure 2:**
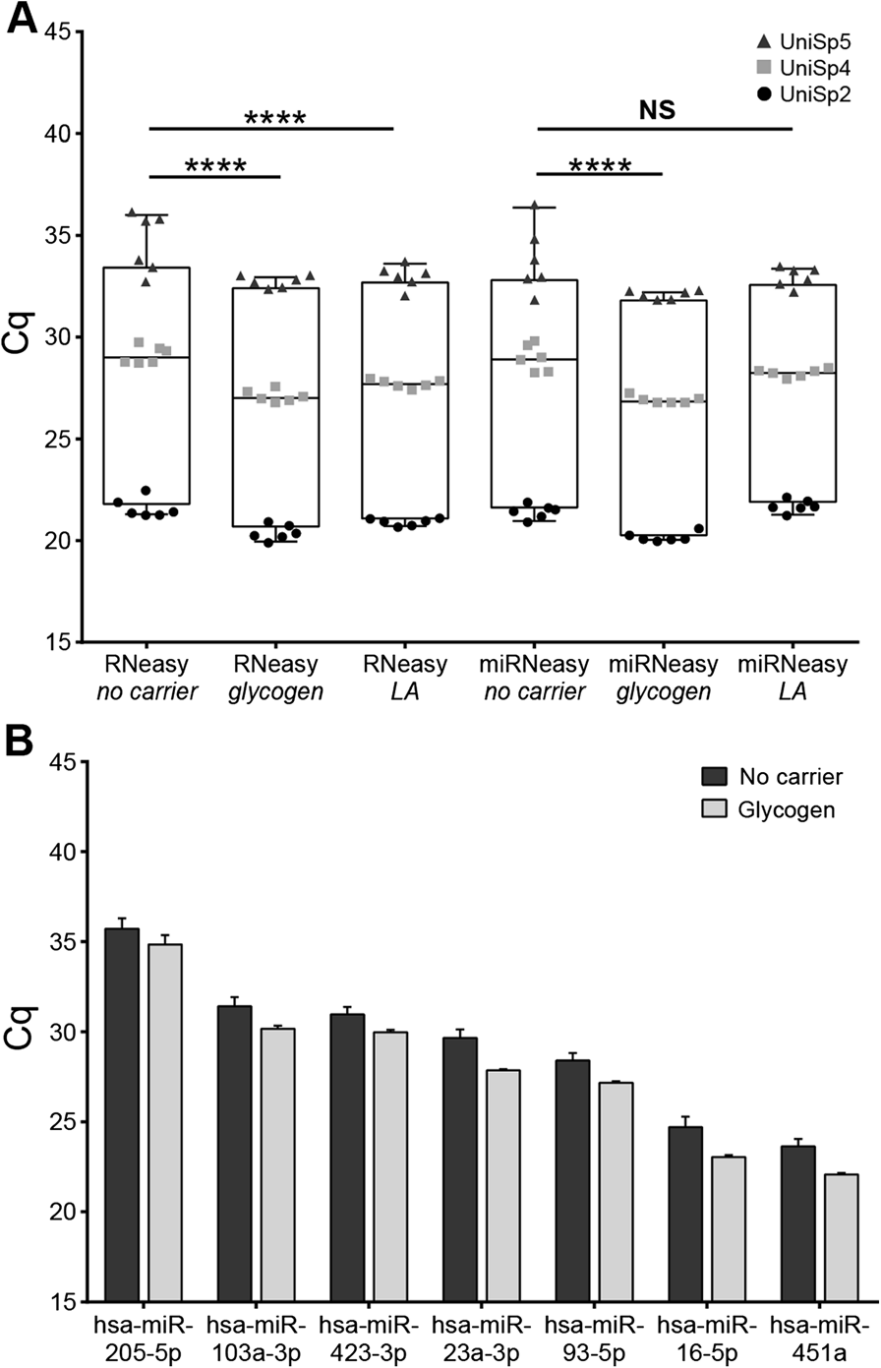
**MiRNA recovery with 5 μg glycogen or linear acrylamide as a co-precipitant during RNA extraction. A**. The effect of glycogen or linear acrylamide (LA) on miRNA recovery was determined for the RNeasy and miRNeasy kits. Boxplot whiskers indicate minimum and maximum values, with the line drawn at the median. Individual datapoints show the spread (n = 6 for each sequence; total n = 18). Extraction efficiencies were compared to the no carrier control sample, and significance was calculated using a linear mixed effects model. **** indicates p < 0.0001, NS = not significant. **B**. The impact of glycogen on endogenous miRNA recovery was assessed using real-time RT-PCR. The error bars represent the standard deviation from six technical replicates.

Glycogen or linear acrylamide is a common co-precipitant for enhanced RNA recovery [3,16,23,24]. Preliminary testing using synthetic spike-in sequences showed that the addition of these carriers improved short RNA recovery (Figure 2A). Linear acrylamide decreased the mean Cq by 1.2 using the RNeasy kit (linear mixed effects model, p < 0.0001) but did not improve Cq values relative to the no carrier control with the miRNeasy kit. Glycogen addition improved detection by 1.7 Cq earlier, on average, for both kits (p < 0.0001). While differences between the kits were not significant, the miRNeasy kit with glycogen showed a trend of decreased variability. The addition of glycogen decreased Cq for all miRNAs tested by an average of 1.3 (p < 0.0001), and appeared to reduce variability for the higher-abundance sequences. Thus the miRNeasy kit, with glycogen carrier, was used for further experimentation using seven endogenous miRNAs (Figure 2B).

### MiRNA extraction efficiency decreases with plasma volume

**Figure 3:**
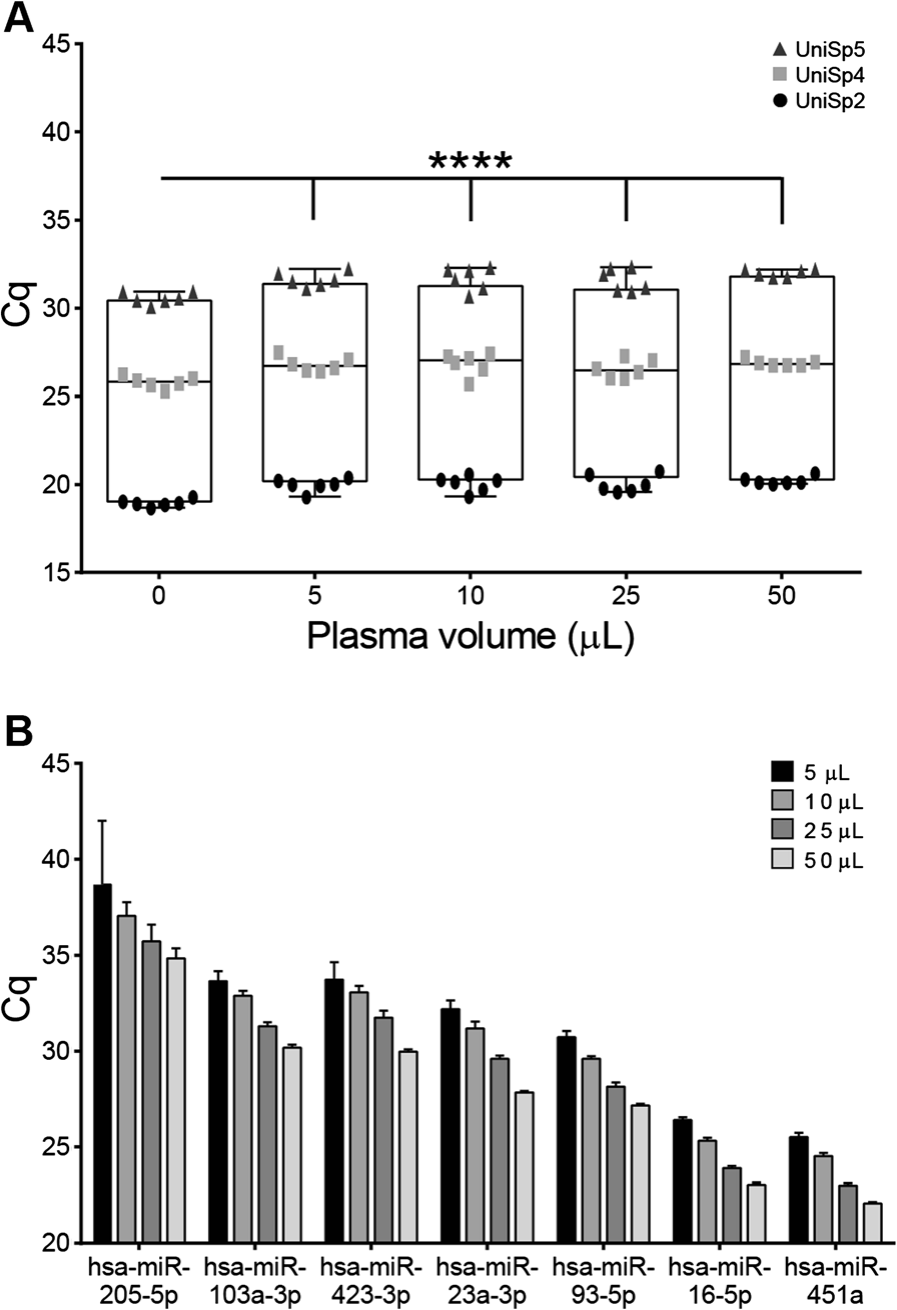
**MiRNA recovery from decreasing amounts of plasma input.** The impact of plasma volume on the recovery of **(A)** exogenous RNA sequences and **(B)** endogenous miRNAs was evaluated using real-time RT-PCR. Plasma samples were diluted in water to 50 μL and processed with the QIAGEN miRNeasy Mini Kit. In **(A)**, three synthetic sequences of different amounts were spiked into the samples and measured in six technical replicates. Significance compared to the water-only control was determined using a linear mixed effects model, **** = p < 0.0001. Endogenous miRNAs were not detected in any water-only controls (Cq > 40).

One significant limitation of studies using clinical specimens (i.e., human or NHP) is restricted sample collection. Here, we evaluated miRNA recovery efficiencies using different starting volumes of NHP plasma to determine the lowest volume that could be used for miRNA profiling. Recovery of both spike-in and endogenous miRNA sequences from a range of input plasma volumes showed significantly higher exogenous RNA expression levels in the water-only control (0 μL plasma, Figure 3A) compared to those obtained from 5, 10, 25, or 50 μL plasma (linear mixed effects model, p < 0.0001).

Expression levels decreased with plasma volume (p < 0.0001) for the endogenous miRNAs assayed (Figure 3B). The water-only (no plasma) negative controls showed no observable contamination, while all seven miRNAs were detected from 5 μL of plasma eluted in 50 μL water after column clean-up. However, PCR technical replicates showed the absence of target in 50% of the lowest abundance hsa-miR-205-5p (mean Cq = 37.41) and in 10% of hsa-miR-423-3p (mean Cq = 33.74). Consequently, subsequent miRNA profiling experiments used 50 μL of plasma.

### Evaluation of miRNA extraction methodology for miRNome profiling using archived plasma from one Ebola virus-challenged NHP

We next evaluated the optimized miRNA extraction protocol using archived rhesus macaque plasma samples from one Ebola virus-infected NHP. MiRNA profiling was conducted on two separate plasma samples from one animal collected at day 0 and day 7 following exposure to aerosolized virus. Using raw Cq values and prior to any normalization steps, 536, 389, 251, and 174 miRNA sequences were detected at Cq cutoffs of 40, 35, 32, and 30, respectively. These sequences were detected in at least one timepoint and in at least one PCR technical replicate.

**Figure 4:**
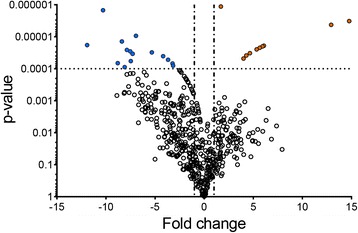
**MiRNA changes in one rhesus macaque following exposure to aerosolized Ebola virus.** Plasma samples from a single NHP challenged with aerosolized Ebola virus were processed using the optimized miRNA extraction protocol. Individual miRNAs were quantified using the Exiqon microRNA Ready-to-Use PCR Human panel I + II V3.R. Twenty-five miRNA sequences (colored filled circles above the horizontal line at p = 0.0001) were significantly altered between virus exposure and time of death. Orange circles indicate upregulated miRNAs, while blue circles specify downregulated sequences. A paired t-test using three PCR technical replicates at each timepoint (day 0 and day 7) was used to identify differentially expressed miRNAs in these datasets.

Further analysis confirmed the presence of 519 of the 752 unique miRNAs (for Cq < 40) included in the Exiqon Ready-to-Use Human panel I + II V3.R in at either day 0 or day 7. Of these, 92 sequences were uniquely present at time of death, while one miRNA was novel to day 0 (data not shown). Overall, a comparison of the miRNA profiles for both days (paired t-test using three technical PCR replicates per timepoint) revealed 25 sequences significantly up- or down-regulated (p < 0.0001) between pre-infection and time of death (Figure 4).

Interestingly, the proportion of plasma miRNA sequences detected (71.3% of the Exiqon panel) parallels the percentage of rhesus macaque sequences that are identical to human (75.5% of miRBase release 12 [25]). As well, sequences reported to be potentially affected by TRIzol isolation [17] were represented in our dataset (Additional file 2: Table S1).

## Discussion

Numerous pre-analytical variables in cell-free blood can significantly impact apparent miRNA profiles. For example, while the miRNA spectra in both plasma and serum are highly correlated [26,27], the use of plasma can avoid clotting-triggered release of miRNAs that can obscure disease-specific sequences [28]. Similarly, for plasma, EDTA is the preferred anticoagulant as heparin can inhibit PCR [29,30] while sodium citrate can dilute plasma and cause hemolysis [31]. Blood samples must also be processed within 1 hour of collection [32], and additional centrifugation is required to produce platelet-poor plasma. Using platelet-depleted plasma substantially decreases blood cell-associated miRNAs, preserves relevant species in circulation, and reduces the variance in expression levels [19]. However, blood cell rupture can also inflate associated miRNA levels so hemolysis must be checked with a hematology analyzer prior to RNA extraction [11], or possibly after PCR by calculating the difference between miRNAs unaffected and affected by hemolysis (Cq(hsa-miR-23a-3p)-Cq(hsa-miR-451a)) [11,21]. Overall, these factors necessitate consistent and high efficiency extraction protocols for reliable profiling of miRNAs in disease states.

Multiple kits and methodologies are currently available for plasma miRNA isolation. However, samples containing category A pathogens must be inactivated, typically using TRIzol LS or similar guanidinium and phenol solutions, prior to removal from biosafety level (BSL)-3 and BSL-4 biocontainment suites [12,13]. While standard RNA extraction from TRIzol can improve miRNA yield and decrease interassay variability compared to column-based kits [10], this method is time-consuming and highly dependent on operator skill. Instead, we chose to optimize TRIzol-treated plasma lysate as the input to five column-based RNA purification kits. Manufacturers’ protocols were modified where necessary to isolate total RNA, as expression profiling using only the small RNA fraction (<200 nt) was shown to be inferior to using total RNA, with 65-88% apparent expression loss after small RNA enrichment [20]. Based on RT-PCR assays for spiked-in and endogenous sequences, the QIAGEN RNeasy and miRNeasy kits performed best. Both enabled miRNA detection 2.0 cycles earlier, on average, than the closest competitor. All kit protocols facilitated quick and easy RNA isolation, but the Zymo Direct-zol kit, which takes plasma-TRIzol lysate without phase separation as input, took significantly less time to complete. We tested this monophase input with the QIAGEN RNeasy and miRNeasy kits to decrease extraction time, but observed greater variability in miRNA expression. We therefore recommend phase separation for miRNA profiling studies.

Plasma contains very low levels of RNA, so carrier molecules such as yeast RNA, glycogen, or linear acrylamide can be included to improve miRNA recovery and extraction reproducibility [33,34]. Results here showed that glycogen was a superior carrier, enhancing miRNA detection ~3-fold compared to extractions with no carrier or with linear acrylamide. Interestingly, linear acrylamide did not improve RNA recovery when used with the QIAGEN miRNeasy kit, but did increase miRNA yield with the RNeasy kit. While the use of biologically-derived glycogen could potentially interfere with downstream analyses [3], no amplification was observed using endogenous miRNA primer sets with no reverse transcriptase controls (data not shown).

We also tested the effect of decreasing the initial plasma amount on miRNA profiling. The use of low plasma volumes is particularly relevant in studies with restricted human or NHP clinical samples. NHP studies are usually limited in animal number due to ethical reasons as well as the costs of care and biocontainment. Repeated or large volume blood collection may also become problematic as the disease progresses. Further, several recent studies have established that input plasma volumes over 200 μL may confound miRNA profiling due to spin column clogging or polymerase inhibitor carryover [16,35,36]. In one study, reducing plasma input volume from 200 μL to 50 μL improved the detection of endogenous hsa-miR-16-5p threefold, supporting the argument for lower sample input volumes [15]. Here, we examined differences in plasma miRNAs for sample volumes from 5 to 50 μL. These results showed that even 5 μL of plasma significantly decreased recovery of exogenous RNA. This may be attributed to RT or PCR inhibition due to the carryover of plasma components, as spin column clogging from such a low sample volume is unlikely, and the low amounts of RNA recovered argue against reagent limitation during cDNA synthesis. Seven plasma miRNAs, spanning a range of abundances, were detected in all sample volumes tested. However, the identification of lower-abundance miRNAs from very low plasma volumes may be compromised. Based on these findings, we recommend miRNA profiling from 50 μL of plasma. Once suitable miRNA biomarkers have been identified and validated, it may be possible to use minimal sample volumes if miRNA abundance is not expected to be low.

As a proof of concept, we used this extraction methodology with archived plasma samples from an unrelated study of NHPs exposed to aerosolized Ebola virus [37]. We chose the LNA-based Exiqon platform because of its demonstrated sensitivity and specificity for clinical samples with low miRNA abundance [38,39]. With a Cq cutoff of 40, 536 (71.3%) of the 752 unique miRNA sequences were detected in at least on PCR replicate and in either timepoint. This number is close to the reported percentage of rhesus macaque sequences that are identical to human miRNAs, which is 75.7% (523 of 692 miRNAs recognized at the time) [25]. The number of miRNAs detected from 50 μL plasma is also comparable to the number of miRNAs identified from 200 μL of starting material assayed with an older version of the Exiqon panel [8]. Both of these results are in contrast to a similar study using 300 μL of pre-amplified plasma, where at most 65 miRNAs (out of 754) profiled with the TaqMan Array Human MicroRNA A+B Cards were recognized [40].

The level of miRNA representation from the Exiqon panels suggests that this method may circumvent TRIzol-associated loss of certain miRNAs. This selective recovery from using TRIzol for miRNA isolation from low numbers of cells was reported for sequences with low GC content and stable secondary structure, such as hsa-miR-141-3p, -193a-3p, -301a-3p, -200a-3p, -15a-5p, -324-5p, -106b-5p, -34a-5p, -21-5p, -20a-5p, -19b-3p, -29b-3p [17]. MiRNAs reportedly unaffected by TRIzol extraction include those from the let-7 family, hsa-miR-200c-3p, -29a-3p, and -25-3p. The authors postulated that small RNAs need to be carried by longer transcripts during the precipitation step of non-column-based TRIzol extraction. Structured, GC-rich miRNAs likely base-pair less efficiently to these longer RNA sequences and are therefore lost during the purification process. Our method aimed to avoid biases in miRNA recovery by adding an RNA carrier and by using spin columns instead of liquid phase precipitation. These findings indicate that our method, and the Exiqon panels, are well-suited for miRNA profiling from low volumes of frozen plasma. In a preliminary investigation using plasma from one rhesus macaque, a comparison of the miRNAs expressed at day 0 and day 7 post-exposure to aerosolized Ebola virus identified 25 differentially expressed sequences. While the biological and statistical relevance of these sequences is under study, the results demonstrate that the miRNA extraction and profiling methods chosen are suitable for potential biomarker discovery. Pending confirmation using archived samples from the same NHP cohort, differentially expressed miRNA sequences may be used to construct an Ebola virus miRNA signature of infection.

## Conclusions

In this work, we present an optimized RNA extraction protocol for TRIzol-inactivated plasma samples. We evaluated five commercially available spin column-based kits for the isolation of total RNA from TRIzol-lysed nonhuman primate (NHP) plasma samples. The QIAGEN miRNeasy Mini Kit, used with the aqueous phase of 50 μL plasma co-purified with 5 μg of glycogen, yielded the highest miRNA recovery. We verified this protocol using archived plasma from one rhesus macaque exposed to aerosolized Ebola virus. Preliminary findings from two plasma collection timepoints indicate that this method can be used for robust miRNA profiling from low sample volumes.

## Methods

### Ethics statement

The rhesus macaque plasma samples were archived samples not collected specifically for this study. The NHP experiment and procedures were approved by the U.S. Army Medical Research Institute of Infectious Diseases (USAMRIID) Institutional Animal Care and Use Committee (IACUC) and were carried out in compliance with the regulations outlined in the USDA Animal Welfare Act (PHS Policy) and other Federal statutes and regulations relating to animals and experiments involving animals. The facility where this research was conducted is accredited by the Association for Assessment and Accreditation of Laboratory Animal Care, International and all animal work done adhere to the conditions specified in the Guide for the Care and Use of Laboratory Animals [41]. Animals were given enrichment (including toys and mirrors) regularly as recommended by the Guide for the Care and Use of Laboratory Animals. Food was provided (commercial biscuits, fruit), and animals were checked at least daily according to the protocol. All efforts were made to minimize painful procedures; the attending veterinarian was consulted regarding painful procedures, and animals were anesthetized prior to phlebotomy. Following the development of clinical signs, animals were checked multiple times daily. When clinical observations and scores of animals reached defined levels based on the approved IACUC protocol (scores based on a combination of responsiveness, recumbency, and clinical signs), animals were euthanized by exsanguination following deep anesthesia and administration of a pentobarbital-based euthanasia solution to minimize pain and distress. All animals were housed at USAMRIID.

### Collection and processing of blood from healthy nonhuman primates

Blood samples were obtained from three healthy male cynomolgus monkeys housed at the USAMRIID Veterinary Medicine Corps. Whole blood (20 mL from each animal) was collected into 10 mL EDTA tubes (BD Biosciences, Franklin Lakes, NJ) and processed within 1 hour, as recommended in the National Cancer Institute Early Detection Research Network consensus statement [32]. Blood tubes were spun in an Eppendorf 5810R centrifuge at 1,300 × *g* for 15 minutes at room temperature, with no brake applied, to obtain plasma. The upper (plasma) phase was collected to above 5 mm of the buffy coat for each sample, and combined to produce one pooled sample for all subsequent steps. Platelet-poor plasma was obtained by distributing the pooled sample into clean 15 mL conical centrifuge tubes and re-spinning at 3,220 × *g* for 10 minutes at room temperature with a brake setting of 9. The supernatant was transferred to 1.5 mL microcentrifuge tubes and stored at -80°C until use. Plasma was analyzed for a complete blood count with a Cell-Dyn 3700 hematology analyzer (Abbott Laboratories, Abbott Park, IL).

Archived plasma samples from rhesus macaques challenged with aerosolized Ebola virus were acquired. Blood samples were obtained with Vacuette K_2_ EDTA tubes. Plasma was separated by centrifugation at 3,000 × g for 10 minutes, mixed with 3 volumes of TRIzol LS (Life Technologies, Grand Island, NY) and archived at −80°C until use. These plasma samples were not collected for the purpose of this study.

### RNA extraction from healthy nonhuman primate plasma using TRIzol LS

All kit evaluations were performed using the same pooled plasma sample from three healthy NHPs. This sample was aliquoted to avoid freeze-thawing cycles. For the initial five kit evaluation, 50 μL volumes (10 replicates) of the initial pooled sample were extracted with each kit. Subsequent tests of phase input, co-precipitant use, and plasma volume used 6 plasma aliquots per treatment group. For example, for RNA carrier evaluation, 6 plasma aliquots were processed for each test condition (glycogen or linear acrylamide), for a total of 12 extractions per QIAGEN kit. For each RNA extraction, 5 to 50 μL once-thawed plasma was diluted with water to a volume of 250 μL, and 750 μL TRIzol LS was added. The solution was vortexed and incubated at room temperature for 5 minutes. Glycogen or linear acrylamide (5 μg, obtained from Life Technologies) was added at this point for samples with co-precipitants. Synthetic sequences (1 μL) from the RNA spike-in kit (Exiqon, Inc., Woburn, MA) were added, followed by 150 μL of chloroform. Each tube was vortexed vigorously for 30 seconds and allowed to sit at room temperature for 3 minutes. Phase separation was achieved by centrifuging the sample for 12,000 × *g* for 15 minutes at 4°C. After centrifugation, 400 μL of the aqueous phase was carefully transferred to a new tube for spin column purification.

### Total RNA purification using spin column-based kits

The following commercially available RNA purification kits were tested in this study: *mir*Vana miRNA Isolation Kit (Ambion, Austin, TX), miRCURY Isolation Kit – Biofluids (Exiqon, Inc.), RNeasy Mini Kit (QIAGEN Inc., Valencia, CA), miRNeasy Mini Kit (QIAGEN Inc.), and Direct-zol RNA MiniPrep Kit (Zymo Research Corp., Irvine, CA). Total RNA was purified from the aqueous phase, with the exception of the Zymo Direct-zol kit, which was used immediately after the addition of the RNA spike-ins. Kit protocols were modified (Additional file 1: Supplementary methods) to obtain total RNA, and RNA was eluted from all columns with 50 μL molecular biology-grade water.

### Individual microRNA PCR assays

All kits used in this section were obtained from Exiqon, Inc. Extracted RNA was reverse-transcribed with the cDNA Synthesis Kit II using 10 μL reaction volumes with 2 μL of input RNA. Following the manufacturer’s protocol, cDNA was diluted 40× and mixed with the ExiLENT SYBR Green master mix and individual microRNA LNA PCR primer sets. Primer sets used were for the synthetic miRNA spike-ins UniSp2, UniSp4, and UniSp5, as well as for the endogenous targets hsa-miR-16-5p, hsa-miR-23a-3p, hsa-miR-93-5p, hsa-miR-103a-3p, hsa-miR-205-5p, hsa-miR-423-3p, and hsa-miR-451a (Table 1). Amplifications were performed in duplicate in clear 96-well plates on a LightCycler 480 II (Roche, San Francisco, CA) using manufacturer-recommended cycling conditions: 95°C for 10 min, 45 cycles of 95°C for 10 s, 60°C for 1 min with a ramping rate of 1.6°C/s, and a final melt-curve analysis.

### RNA extraction and analysis of microRNA content in Ebola virus-infected nonhuman primates

Archived plasma samples from one rhesus macaque, collected at days 0 and 7 (time of death) post-challenge, were thawed on ice. The animal was anesthetized with 6 mg/kg telazol prior to phlebotomy on both day 0 and day 7. Blood collection on day 7 was performed ante-mortem, after administration of telazol but before pentobarbital. Two hundred (200) μL TRIzol-plasma lysate (containing 50 μL plasma) was processed with 5 μg glycogen as described for the healthy nonhuman primate plasma samples. After phase separation, total RNA was purified from 400 μL of the aqueous phase using the modified miRNeasy kit protocol (Additional file 1: Supplementary methods). Extracted RNA was reverse-transcribed with the cDNA Synthesis Kit II using 8 μL RNA for each 40 μL sample. cDNA samples were pooled and mixed with ExiLENT SYBR Green master mix, and 10 μL of this mixture was used in each well of the microRNA Ready-to-Use PCR Human panel I + II V3.R (Exiqon Inc.). Three PCR technical replicates were run for each time point, using the same cycling conditions described above for individual miRNA assays.

### Data analyses

Cq values for individual assays were obtained using the second derivative method within the integrated Roche LightCycler 480 software version 1.5.1. A sample was considered positive if Cq < 40, and missing Cq values (from undetected miRNAs) were set to 45 (total number of PCR cycles) prior to statistical testing. Analyses of RNA extraction parameters were performed in R version 3.1.0 [42], using a linear mixed effects model with the kit as a fixed effect.

GenEx 6 Enterprise software (MultiD Analyses AB, Göteborg, Sweden) was used to analyze PCR panel data. MiRNA expression levels were calculated using the ΔΔCq method [43] with global normalization [44]. Briefly, interplate and spike-in calibrations were performed, followed by removal of samples with Cq > 39.4 and miRNAs that were not detected in 5 of 6 replicates. Global normalization of Cq values was performed by using the mean of all samples with Cq < 36 (per day). Relative quantities of each miRNA were calculated with respect to the average expression level on day 0, then data were log_2_ transformed and compared using a paired t-test. The miRNA RT-PCR data used in this study is available in the Gene Expression Omnibus database (GSE64579).

## Additional files

Additional file 1:
**Supplementary methods.** Manufacturer kit protocols (except for the Zymo Direct-zol protocol) were modified for the isolation of total RNA from plasma. Additional file 2: Table S1. Normalized Cq values of microRNAs (miRNAs) potentially affected/unaffected by TRIzol extraction from low numbers of cells.

## Abbreviations

BSL: Biosafety Level
Cq: quantification cycle
K_2_ EDTA: Dipotassium ethylenediaminetetraacetic acid
hsa-miR: *Homo sapiens* microRNA
IACUC: Institutional Animal Care and Use Committee
LNA: Locked nucleic acid
miRNA: microRNA
NHP: Nonhuman primate
Nt: nucleotide
RNA: Ribonucleic acid
RT-PCR: Reverse transcription polymerase chain reaction
USAMRIID: U.S. Army Medical Research Institute of Infectious Diseases
USDA: U.S. Department of Agriculture
