# Supplementary material for: Optimized microRNA purification from TRIzol-treated plasma
Source: BMC Genomics. 2015 Feb 18;16(1):95. doi: 10.1186/s12864-015-1299-5 (PMC4342875; doi:10.1186/s12864-015-1299-5)
Supplement: Additional file 1: — Supplementary methods. Manufacturer kit protocols (except for the Zymo Direct-zol protocol) were modified for the isolation of total RNA from plasma. [file 12864_2015_1299_MOESM1_ESM.pdf]

Supplementary Methods. Manufacturer kit protocols (except for the Zymo Direct-zol protocol) were modified as follows for the isolation of total RNA from plasma. The aqueous phase is used as input to the kits.

Ambion *miR*vana miRNA Isolation Kit

Preheat nuclease-free water to 95°C for use in eluting the RNA from the filter at the end of the procedure.

100% ethanol must be at room temperature

1) Add **1.25 volumes of room temperature 100% ethanol** to the aqueous phase.
2) **Pass the lysate/ethanol mixture through a Filter Cartridge**
    a. For each sample, place a Filter Cartridge into one of the Collection Tubes supplied.
    b. Pipet the lysate/ethanol mixture (from the previous step) onto the Filter Cartridge. Up to 700 µL can be applied to a Filter Cartridge at a time, for samples larger than this, apply the mixture in successive applications to the same filter.
    c. Centrifuge for ~15 sec to pass the mixture through the filter. Centrifuge at **10,000 x g**. Spinning harder than this may damage the filters. Discard the flow-through, and repeat until all of the lysate/ethanol mixture is through the filter. Reuse the Collection Tube for the washing steps.
3) Apply **700 µL miRNA Wash Solution 1** (working solution mixed with ethanol) to the Filter Cartridge and centrifuge for ~5–10 sec or use a vacuum to pull the solution through the filter. Discard the flow-through from the Collection Tube, and replace the Filter Cartridge into the same Collection Tube.
4) **Wash the filter twice with 500 µL Wash Solution 2/3**
    a. Apply 500 µL Wash Solution 2/3 (working solution mixed with ethanol) and draw it through the Filter Cartridge as in the previous step.
    b. Repeat with a second 500 µL aliquot of Wash Solution 2/3.
    c. After discarding the flow-through from the last wash, replace the Filter Cartridge in the same Collection Tube and spin the assembly for 1 min to remove residual fluid from the filter.
5) Transfer the Filter Cartridge into a fresh Collection Tube (provided with the kit). Apply **50 µL of pre-heated (95°C) nuclease-free water** to the center of the filter, and close the cap. Spin for ~20–30 sec at maximum speed to recover the RNA.

Exiqon miRCURY RNA Isolation Kit – Biofluids

1) Add **1 volume isopropanol**, vortex for 5 s.
2) Place a **microRNA Mini Spin Column BF** in a collection tube and load sample onto column. Incubate for 2 min at room temperature. Centrifuge for 30 s at **11,000 x g**.

Discard flow-through and place column back into collection tube. Repeat until all of the sample has been spun through the column.

3) Add **100 µL Wash Solution 1 BF** to the microRNA spin column BF. Centrifuge for 30 s at 11,000 x g. Discard flow-through and place column back into the collection tube.
4) Add **700 µL Wash Solution 2 BF** to the microRNA spin column BF. Centrifuge for 30 s at 11,000 x g. Discard flow-through and place column back into the collection tube.
5) Add **250 µL Wash Solution 2 BF** to the microRNA spin column BF. Centrifuge for 2 min at 11,000 x g to dry the membrane completely.
6) Place the microRNA spin column BF into a new 1.5 mL collection tube. Add **50 µL RNase-free water** directly onto the membrane of the microRNA spin column BF. Incubate for 1 min at room temperature, then close the lid and centrifuge for 1 min at 11,000 x g.

<u>QIAGEN RNeasy MinElute</u>

From Qiagen supplementary protocol [39]

1) Add **1.5 volumes of 100% EtOH** to the recovered aqueous phase, mix thoroughly by vortexing. Do not centrifuge. Proceed immediately to next step.
2) Load **700 µL** of sample, including any precipitate, onto an **RNeasy Mini spin column** in a 2 mL collection tube. Close lid gently and spin for **15 s** at **8000 x g**. Discard flow-through and repeat step until all sample has been loaded.
3) Transfer column to a new collection tube, add **500 µL buffer RPE** to the column and spin for 15 s at 8000 x g to wash the column membrane. Discard flow-through.
4) Add **500 µL buffer RPE** to the spin column. Close lid gently and centrifuge for 15 s at **8000 x g** to wash the column membrane. Discard flow-through and collection tube.
5) Place column into a new collection tube. Close the lid and centrifuge at full speed for 1 min.
6) Transfer column to a clean 1.5 mL collection tube and add **50 µL RNase-free water** to the center of the column membrane. Allow the sample to sit for 1-2 min at room temperature, then spin for 1 min at **8000 x g** to elute RNA.

QIAGEN miRNeasy Mini Kit

From Qiagen supplementary protocol [40]

1) Add **1.5 volumes of 100% EtOH** and mix thoroughly by pipetting up and down several times. Do not centrifuge. Proceed immediately to next step.
2) Transfer up to 700 µL of sample, including any precipitate, into an **RNeasy Mini spin column** in a 2 mL collection tube. Close the lid gently and centrifuge for 15 s at **8000 x g** at room temperature. Discard flow-through, and repeat step until all of the sample has passed through the column.
3) Add **700 µL buffer RWT** to the RNeasy Mini spin column. Close the lid gently and centrifuge for 15 s at 8000 x g to wash the column. Discard flow-through.
4) Add **500 µL buffer RPE** to the column. Close the lid gently and centrifuge for 15 s at 8000 x g to wash the column. Discard the flow-through.
5) Repeat the preceding wash step.
6) Place the column into a new collection tube (avoid ethanol carryover from previous collection tube). Centrifuge at full speed for 2 min to dry the spin column membrane.
7) Transfer the column into a clean 1.5 mL tube. Add **50 µL of RNase-free water** directly to the membrane. Close the lid gently and centrifuge for 1 min at 8000 x g to elute the RNA.
