# Supplementary material for: Optimized microRNA purification from TRIzol-treated plasma
Source: BMC Genomics. 2015 Feb 18;16(1):95. doi: 10.1186/s12864-015-1299-5 (PMC4342875; doi:10.1186/s12864-015-1299-5)
Supplement: Additional file 2: Table S1. — Normalized Cq values of microRNAs (miRNAs) potentially affected/unaffected by TRIzol extraction from low numbers of cells. [file 12864_2015_1299_MOESM2_ESM.pdf]

Supplementary Table. Normalized Cq values of microRNAs (miRNAs) potentially affected/unaffected by TRIzol extraction from low numbers of cells. Putatively affected sequences given here are characterized by low GC content and stable secondary structure [1].

microRNA (miRBase 21 nomenclature)	Cq values at Day 0	Cq values at Day 7	
hsa-miR-141-3p	34.62	31.54	Potentially affected by TRIzol extraction
hsa-miR-21-5p	28.38	19.19	
hsa-miR-106b-5p	30.41	25.89	
hsa-miR-15a-5p	29.30	23.58	
hsa-miR-34a-5p	32.76	27.09	
hsa-miR-193a-3p	37.97	28.50	
hsa-miR-301a-3p	34.24	30.08	
hsa-miR-200a-3p	Not detected	32.31	
hsa-miR-324-5p	37.17	29.81	
hsa-miR-20a-5p	28.67	24.19	
hsa-miR-19b-3p	27.52	21.52	
hsa-miR-29b-3p	33.57	26.36	
hsa-miR-200c-3p	36.58	33.05	Unaffected
hsa-miR-29a-3p	31.67	22.59	
hsa-miR-25-3p	30.92	24.66	

Reference

1. Kim Y-K, Yeo J, Kim B, Ha M, Kim VN: **Short Structured RNAs with Low GC Content Are Selectively Lost during Extraction from a Small Number of Cells.** *Molecular Cell* 2012, **46:**893-895.
